# Supplementary material for: Practice and factors associated with pediatrics pain management among nurses working in Bahir Dar city public hospitals: A mixed method study
Source: PLoS One. 2024 May 6;19(5):e0300853. doi: 10.1371/journal.pone.0300853 (PMC11073712; doi:10.1371/journal.pone.0300853)
Supplement: S1 File — (PDF) [file pone.0300853.s001.pdf]

Quantitative Questionnaire

Pain management practice for hospitalized children's

Date______________ unit code _______________

Instructions: Read each question carefully and tick or circle against the option that best suits your response.

Part I: Socio-demographic-related questions

S. No	Question	Response	Remark
101	Age (in years)	--------------	
102	Sex	1. Male<br>2. Female	
103	How many years of experience	1. < 2yrs 2 .2-5 yrs.<br>3. 5-10yrs 4.>10yrs	
104	Educational status	1. Diploma<br>2. BSc in comprehensive nurse<br>3. BSc in pediatrics and child health nursing<br>4. BSc neonatal nurse<br>5. MSc and above<br>6. Others……………	
105	Which ward are you currently giving service?	1. Medical ward<br>2. Surgical ward<br>3. Pediatric ward<br>4. Emergency/ETAT<br>5. ICU/NICU<br>6. Orthopedic ward<br>7. Others--------	

106	How many months /years due you work in pediatrics and child health wards/ETAT?	-----------------	
108	Monthly income (in ETB)	-------------------	

Part II: Questions related to Practice of nurses for pain management

No,	Items	Yes	No
**201**	Do you assess pain in children?		
**202**	If yes, do you use a pain assessment tool? (If no, please go to question number 204)		
**203**	If yes, do you use a pain assessment tool for patients routinely?		
**204**	Do you use a self-reported pain scale (FACE scale) for assessment of children's pain in your practice?		
**205**	Do you use a behavioral pain scale (FLACC) for assessment of children's pain in your practice?		
**206**	Do you administer pain medication to children by your adjustment?		
**207**	Do you administer additional pain medication to relieve pain when needed or (PRN?)		
**208**	Do you reassess children's pain after giving pain medication to evaluate the effectiveness of the pain medication?		
**209**	Do you give post-operative analgesics around the clock on a fixed schedule?		
**210**	Do you give children sterile water by injection (placebo) to determine if the pain is real?		
**211**	After the initial recommended dose of Opioid analgesic, do you adjust subsequent doses to the individual /patient's response?		
**212**	Do you check and report for seniors if you find skin rash, headache, vomiting, and increased heart rate after administering Opioid analgesics		
**213**	Do you document the findings after pain assessment and management for patients?		
**214**	Do you assess and administer analgesia before the following procedures are done? **Say "Yes or No"**		

	a. Patient repositioning		
	b. End tracheal suctioning<br>c. Wound care		
	d. pre-and post-operatively		
**215**	Do you discuss pain scores and management" during nurse-to-nurse reports?		
**216**	Are pain scores and management discussed during unit rounds?		
**217**	Do you always agree with children's statements about their pain?		
**218**	Do you use non-pharmacological intervention for children's pain management (If your answer is **yes** go to the next section)		
**219**	If yes, which methods do you use for children's pain? **Say "Yes or No"**		
	1. Hot/Cold, compress		
	2. Music		
	3. Playroom		
	4. Pressure		
	5. prayer		

PART III: Questions related to nurses' knowledge of pediatrics pain management

301. 'Thick or circle' for medications you know below that are mostly available in your institution for pain relief measures? More than one answer is possible.

1. Paracetamol 2. Ibuprofen 3. Diclofenac

4. Tramadol 5. Morphine 6. Fentanyl

302. Which drug is used to treat moderate and severe pain in the pediatric age group? More than one answer is possible.

1. Paracetamol 2. Ibuprofen 3. Diclofenac

4. Tramadol 5. Morphine 6. Fentanyl

303. Is it important to manage pain for the following classifications of patients?

a. post-operative patient 1. Yes, 2. No

b. Medical (nonsurgical) patients 1. Yes, 2. No

c. Patients with a Glasgow Coma Scale < 8 1. Yes 2. No

d. Trauma patients 1. Yes, 2. No

e. Burn patients 1. Yes, 2. No

f. End-of-life patients 1. Yes, 2. No

304. Do you think it is important to manage pain and the need for analgesia before, during, and after the following procedures?

a. Patient repositioning 1. Yes, 2. No

b. Endotracheal suctioning 1. Yes, 2. No

c. Wound care 1. Yes, 2. No

d. Drain removal 1. Yes, 2. No

e. Postoperatively 1. Yes, 2. No

f. Securing IV lines 1. Yes, 2. No

305.The recommended route of administration of Opioid analgesics to children with brief, severe pain of sudden onset (e.g. trauma or postoperative) pain is:

a. Intravenous c. Intra muscular

b. Oral d. Intra dermal

306. Giving narcotics on a regular schedule is preferred over a PRN schedule for continuous pain. 1. Yes 2. No

307. Lack of pain expression does not mean lack of pain. 1. Yes 2. No

308. Distraction, for example, by the use of music or relaxation, can decrease the feeling of pain.
1. Yes 2. No

309. Increasing analgesic requirements are signs that the patient is becoming addicted to the narcotic. 1. Yes 2. No

310. Patients having severe chronic pain often need higher dosages of pain Medications than patients with acute pain. 1. Yes 2. No

311. If a patient (and/or family member) reports that a narcotic is causing Euphoria, she should be given a lower dose of the analgesic 1. Yes, 2. No

312. Do you know the consequences of unrelieved/ untreated pain? 1. Yes 2. No

313. Narcotics can cause respiratory depression; therefore, they should not be used in pediatric patients? 1. Yes 2. No

314. Is it important to give analgesics for the following acute or chronic cases?

a. Severe headache 1. Yes 2. No

b. Sudden abdominal cramps 1. Yes 2. No

c. chronic joint/muscle pain 1. Yes 2. No

d. Appendicitis 1. Yes 2. No

e. Cancer pain 1. Yes 2. No

f. any Infection 1. Yes 2. No

Part IV: Questions related to the attitude of nurses toward pain management

S. no	Item	Strongly agree	Agree	Not sure	Disagree	Strongly disagree
**401**	Infants and children experience pain equal to that experienced by adults.					
**402**	Children need better attention than adults to manage their pains.					
**403**	Parents should be present during painful procedures.					
**404**	Pain management and pain relief are priorities in children's treatment.					
**405**	Children have the right to appropriate assessment and management of their pain.					
**406**	The most accurate judge of the intensity of the children's pain is patients.					
**407**	For a better assessment of a child's pain, the nurse can discuss it with her/his parents.					
**408**	Assessment and control of child pain led to improvement in his/her parent's satisfaction.					
**409**	Failure to assess and manage the child's pain affects his body and mind in the long term.					

S. no	Item	Strongly agree	Agree	Not sure	Disagree	Strongly disagree
**410**	The nurse’s physical and mental fatigue can affect children's pain relief.					
**411**	Ensuring patient comfort and pain relief is one of the most important tasks of nurses.					
**412**	Communicating with and educating a child’s parent plays an effective role in relieving pain.					
**413**	Nurses can provide the most accurate rating of pain intensity and manage pain.					
**414**	Evaluation and measurement of a child’s pain should be considered as one of the vital signs when examining the child					
**415**	Measurement and control of pain in children leads to improve quality of the child’s life					

Part VI: Questions related to organizational factors for proper pain management

501. Do you have specific protocols for pediatric pain management in your institution?

1. Yes 2. No

502. Do you gate in-service training on pain management?

1. Yes 2. No

503. Please indicate whether an item affects your ability to manage the pain of children's properly **"ticking or circle on yes or no"**

A. availability of anti-pain drug	1. Yes	2. No
B. Nursing workload	1. Yes	2. No
C. Lack of availability of pain assessment tools	1. Yes	2. No
D. Lack of training	1. Yes	2. No
E. Lack of protocols for pain management	1. Yes	2. No
F. Low priority of pain management by unit team	1. Yes	2. No
G. No designated area and material for managing pain	1. Yes	2. No

Part VII: Questions related to patient-related factors for proper pain management

601. Do you think the Patient's inability to communicate has an impact on pain management?

1. Yes 2. No

602. Do you think children’s consciousness level has an impact on pain management? 1. Yes 2. No

603. Do you think being a child (age) has an impact on the management of pain?

1. Yes 2. No

604. Do you think language differences have an impact on the management of pain?

1. Yes 2. No

605. Do you think Parents affect your ability to manage the pain of children properly during pain management practice? 1. Yes 2. No

606. Do you face problems during pain management of the child due to family/ patients’ needs for drugs wrongly? 1. Yes 2. No

607. Do you face problems during pain management for the child due to cultural beliefs?

1. Yes 2. No

608. Do you face problems during pain management of the child due to awareness of families or Childs?

1. Yes 2. No

609. Do children cooperate in managing pain that have history of pain management before? 1. Yes 2. No

Thank you for your participations!
