# Supplementary material for: Practice and factors associated with pediatrics pain management among nurses working in Bahir Dar city public hospitals: A mixed method study
Source: PLoS One. 2024 May 6;19(5):e0300853. doi: 10.1371/journal.pone.0300853 (PMC11073712; doi:10.1371/journal.pone.0300853)
Supplement: S2 File — (PDF) [file pone.0300853.s002.pdf]

Qualitative Questionnaire

1.1. Socio-demographic questions

1. Unit, Hospital: ___________
2. Age: _____
3. Sex: _____
4. Total Years of Experience ……………………
5. Years of unit Experience ………………………
6. Educational Preparation in Nursing
    - o Diploma nursing
    - o BSc comprehensive Nurse
    - o BSc in Pediatrics and child health nurses
    - o BSc in neonatal nurses
    - o Masters in nursing specialties
    - o Others specify ……….

1.2 Main Questions

1. How do you describe the General overview of children's pain in the unit? (Probes: tell your story interims of severity, magnitude, complication, and others you observed in the unit?)
2. How do you assess children's pain complaints in this unit?
3. Are there pain assessment Obstacles, please, tell your story about how to assess children's pain effectively. (Pain Assessment-Related Barriers)
4. To what extent are you satisfied with the assessment of children's pain in this unit?
5. How do you manage children's pain in this unit? (Probes: what are those activities to perform?)
6. Please tell your story about- facing any factors to practice appropriate pain management for hospitalized children. (Probes: what are those Management barriers? Can you explain this a little further?)
7. To what extent are you satisfied with the management of children's pain in this unit?
8. What activities are to be expected from nurses and other concerned bodies to practice appropriate pain management? (Probes: Can you explain this a little further?)
