# Supplementary material for: Practice and factors associated with pediatrics pain management among nurses working in Bahir Dar city public hospitals: A mixed method study
Source: PLoS One. 2024 May 6;19(5):e0300853. doi: 10.1371/journal.pone.0300853 (PMC11073712; doi:10.1371/journal.pone.0300853)
Supplement: Attachment — Submitted filename: Response to Reviewers.docx [file pone.0300853.s003.docx]

**Date: February 24, 2024**

**Rebuttal Letter**

PONE-D-23-42900

Pediatrics Pain Management Practice and Factors Associated with Hospitalized Children among Nurses Working in Bahir Dar City Public Hospitals, Northwest Ethiopia, Mixed Method

PLOS ONE

**Zeamanuel Anteneh Yigzaw**

**To Plos one**

**Dear all,**

We the authors of this manuscript are pleased to thank the journal editors and the reviewers for revising the manuscript and giving your valuable and constructive comments and suggestions that help to improve the manuscript. We have made a rigorous revision of the manuscript as per your questions and comments. We have included the point-by-point response in the table below framed as editors’ comments/questions and authors’ responses. **We have made a severe revision on the entire manuscript that we believe had merit in improving the manuscript and attached it as tracked change and clean version separately**. We are happy to receive additional revisions if any that would have merit in improving the manuscript.

| **Review Comments to the Author** | **Authors Response** |
| --- | --- |
| - Title: the title is too long and it could be most clear for readers. Suggestion: "Practice and Factors Associated with Pediatrics Pain Management among Nurses Working in Bahir Dar City Public Hospitals: a mixed method study"; | **Thank you very much**  Corrected according to the comment given |
| - Introduction: this section is too long. It is suggested that the main topics related to the theme developed in the study should be exposed. It is suggested that lines 69-92 from page 4 and line 98 -107 from page 5 should be removed, once there is a subjective information not directly related to the theme; | **Thank you very much**  Corrected according to the comment given |
| - Materials and Methods: On this topic, there is also superficial information that must be removed. Information about the cities presented on lines 151-165 from page 7-8, is not necessary to be written in this paper. In this same way, details about the sample size calculator explained on page 8, is not necessary to be in this section; | **Thank you very much**  Corrected according to the comments given |

**Thank you very much**
